# Development of a Mesothelin-Binding Engineered Scaffold Protein as a Theranostic for Pleural Mesothelioma

**DOI:** 10.1021/acs.bioconjchem.5c00425

**Published:** 2025-10-27

**Authors:** Roberto Silvestri, Margherita Piccardi, Alessia Laurenza, Filomena Rea, Allison R. Sirois, Martina Lari, Francesco Bartoli, Giovanni Signore, Lorena Tedeschi, Elisabetta Ferraro, Paola Anna Erba, Roberto Giovannoni, Stefano Landi, Federica Gemignani, Sarah J. Moore

**Affiliations:** † Department of Biology, 9310 University of Pisa, Pisa 56126, Italy; ‡ Picker Engineering Program, Smith College, Northampton, Massachusetts 01063, United States; § Molecular and Cellular Biology Program, University of Massachusetts Amherst, Amherst, Massachusetts 01003, United States; ∥ Department of Translational Research and New Technologies in Medicine and Surgery, University of Pisa, Pisa 56126, Italy; ⊥ School of Medicine and Surgery, 9305 University of Milan Bicocca, Milan 20126, Italy; # Institute of Clinical Physiology, CNR, Pisa 56124, Italy

## Abstract

Mesothelin (MSLN) is a tumor biomarker expressed at high levels on the surface of numerous cancers with extremely limited expression in healthy tissues. MSLN-targeting agents developed for diagnosis and therapy could have a significant impact on the management of MSLN-expressing cancers. Pleural mesothelioma (PM) is a deadly cancer that arises from mesothelial cells lining the pleura and is predominantly linked to asbestos exposure. There are currently no effective treatments, and diagnosis occurs in late stages of disease due to the lack of clinical symptoms in the early stages. Recent efforts to diagnose and treat PM have focused on identifying and targeting relevant biomarkers, including MSLN. We engineered proteins based on the nonantibody fibronectin type III (Fn3) protein scaffold that bind MSLN with high affinity and specificity, using yeast-surface display and directed evolution. Previous work with Fn3 scaffold proteins has demonstrated tissue distribution desirable for applications in molecular imaging and targeted radiotherapy, which may overcome limitations encountered thus far with antibody-based approaches to treat PM. The MSLN-targeting Fn3 was further developed for bioconjugation with the 1,4,7,10-tetraazacyclododecane,1-(glutaric acid)-4,7,10-triacetic acid (DOTAGA) radiometal chelator. MSLN-binding Fn3 specifically binds to the MSLN-expressing PM lines, colocalizes with MSLN, and internalizes upon binding. Fn3-DOTAGA was further coupled with cold metal gallium-69, and the resulting conjugate maintained binding with high affinity to MSLN-expressing PM cells. MSLN-binding Fn3-DOTAGA-^69^Ga is a promising molecule with diagnostic and therapeutic relevance, toward applications in molecular imaging and targeted radiotherapy.

## Graphical abstract

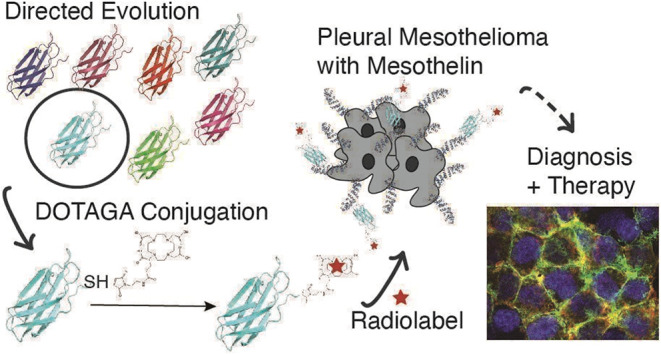

## Introduction

Due to its overexpression in various tumor types and the preferential location at the membrane site, mesothelin (MSLN) represents an attractive molecule for targeted therapies.^1,2^ In addition, MSLN has a role in cancer cell adhesion and metastasis.^3,4^ A recent analysis of more than 12,000 tumors identified various cancer types that might be particularly well suited for anti-MSLN drugs, such as ovarian carcinoma, mesothelioma, and adenocarcinomas of the pancreas, lung, stomach, esophagus, and the colorectum.^5^

Among these, pleural mesothelioma (PM) is an aggressive cancer arising from the mesothelial cells lining the pleura, and it is predominantly associated with asbestos exposure.^6^ Despite its rarity, PM is a fatal cancer accounting for 26,278 new deaths out of 30,870 new cases in 2020.^7^ Diagnosis usually occurs 30–50 years after asbestos exposure and in late stages because clinical signs are not evident in early disease.^6^ Additionally, PM is known to develop resistance to treatments, and surgery is available only for candidate PM cases.^8,9^ Therefore, PM therapy and early diagnosis are still a challenge, leading to poor prognosis with an 8–14 months median survival for PM patients.^10^

In recent decades, measuring MSLN serum levels and applying MSLN-targeted therapies have been proposed to overcome PM challenges.^11,12^ Despite initial enthusiasm, success has been elusive using antibody–drug conjugates directed at the MSLN for PM. A phase II clinical trial with antibody–drug conjugate anetumab ravtansine (BAY 94-9343) targeting MSLN in PM patients was not superior to treatment with chemotherapeutic vinorelbine, although the antibody–drug conjugate did have a clinically manageable safety profile.^13^ Similarly, clinical trials with amatuximab, originally referred to as MORAb-009, have yet to translate into any approved MSLN-targeted therapy.^14–16^ Efforts to improve amatuximab and its conjugates to achieve desired clinical outcomes are ongoing.^16–19^ While multiple CAR T-cell therapies have been developed targeting MSLN, they have also encountered barriers to success.^20–23^ Shedding of MSLN from the cell surface has been proposed as one key barrier to overcome for therapies based on antibody and CAR T-cells,^24,25^ which require extended engagement of the therapy at the cancer cell surface to be effective. Recent reports describe MSLN-targeting antibodies and CAR T-cell formats that recognize a domain of MSLN adjacent to the cell membrane and that is not shed from the cell, which may be able to overcome some of the challenges for these classes of therapy.^26,27^

MSLN-targeting molecules can also be used as noninvasive molecular imaging agents for diagnosis and prognosis, including as a theranostic, with both a therapeutic and diagnostic role. In recent years, a number of antibodies that bind MSLN have been used to validate imaging of MSLN in this patient population using single-photon emission computed tomography (SPECT) and positron emission tomography (PET).^28–31^ Nanobodies that bind MSLN, based on a camelid antibody structure, have been reported for molecular imaging of MSLN in murine xenograft models, with moderate tumor uptake reported.^32,33^ These imaging studies in living subjects demonstrate the utility of molecular imaging of MSLN, while demonstrating a need for developing a new generation of imaging agents with rapid clearance from nontarget tissues and high tumor-to-background ratios.

Mechanistic modeling and experimental evidence suggest that protein scaffolds that are alternatives to antibodies have promise in specific applications where antibody-based approaches encounter challenges. Targeted radionuclide therapy using antibodies as radiolabeled molecules exhibits dose-limiting toxicities due to the long circulation times of antibodies, while using antibody fragments or alternative protein scaffolds can address these limitations.^34^ Diagnostic molecular imaging can also benefit from small alternative protein scaffolds, where rapid clearance from circulation and nontarget tissues is desired alongside rapid accumulation in targeted tissues to achieve high signal-to-background ratios.^35–40^ High-affinity, smaller protein structures can be beneficial for allowing targeting molecules to reach throughout a dense solid tumor, including for protein–drug conjugates, to reduce off-target toxicity from long circulation times and avoid therapeutic resistance that can occur when antibodies and antibody–drug conjugates fail to reach the center of tumors.^41–43^ A variety of protein structures that can be engineered as binding molecules have been developed, including affibodies, DARPins, fibronectin type III (Fn3s), nanobodies, knottins, and the Gp2 fold.^44–50^ Such protein structures can be modified through a variety of approaches to tailor their pharmacokinetics, such as through reformatting as Fc fusions, expressing with an albumin binding peptide domain, or conjugating to a polymer such as polyethylene glycol or other half-life extension tags, depending on the design considerations for the specific application.^51–57^

Recently, we have reported initial work to engineer the Fn3 alternative scaffold as a theranostic molecule for MSLN-positive tumors, using directed evolution with yeast-surface display.^58,59^ We previously demonstrated that our early generations of engineered Fn3 molecules bound specifically to MSLN-positive cell lines, were internalized, and made MSLN-positive cancer cell lines more sensitive to traditional chemotherapy. Here, we report a new generation of MSLN-binding Fn3 molecules with low-nanomolar dissociation constants (*K*_D_) and their application as promising theranostic agents of MSLN-positive PM. We report methods for coupling the engineered Fn3 variants with ^69^Ga by 1,4,7,10-tetraazacyclododecane,1-(glutaric acid)-4,7,10-triacetic acid (DOTAGA) conjugation and radiometal chelation and validate their use in PM cell lines. These molecules are promising candidates for continued development in preclinical models of PM toward the goal of better ways to diagnose and treat PM and other MSLN-positive tumors.

## Results and Discussion

### Fn3 Protein Variant 5.3.2 Binds MSLN with High Affinity

Previously, we reported engineering Fn3 variants that bind MSLN with dissociation constants (*K*_D_) with moderate nanomolar (nM) affinities, and demonstrated that these variants were internalized and had a cytotoxic effect on MSLN-positive tumor cell lines.^58,59^ Toward the goal of improved binding affinities beneficial for clinical translation with *in vivo* applications, we continued with affinity maturation of the third-generation Fn3 library (Figure 1). Our previously reported MSLN-binding Fn3 library was further mutated and affinity matured as fourth- and fifth-generation libraries using yeast-surface display and directed evolution. The resultant population yielded the enrichment of evident MSLN-binding clones. Sequence analysis of individual clones identified two dominant variants, 5.3.1 (20% of sequenced variants) and 5.3.2 (25% of sequenced variants) (Figure 1C), in addition to 11 other sequences with low representation in the sequenced library. All unique variants were tested for binding to Fc-MSLN using YSD with a single concentration of Fc-MSLN, and 5.3.1 and 5.3.2 exhibited greater binding signals compared with all other variants tested (data not shown). Of isolated sequences, variant 5.3.2 demonstrated the highest affinity to Fc-MSLN based on equilibrium binding assays of enriched Fn3 variants analyzed from all completed rounds of affinity maturation (Figure 1C) and exhibited high recombinant expression and ease of purification (Figure 1D). Therefore, we pursued Fn3 5.3.2 for additional characterization and bioconjugate modification toward nuclear medicine applications, particularly for PM.

**Figure 1:**
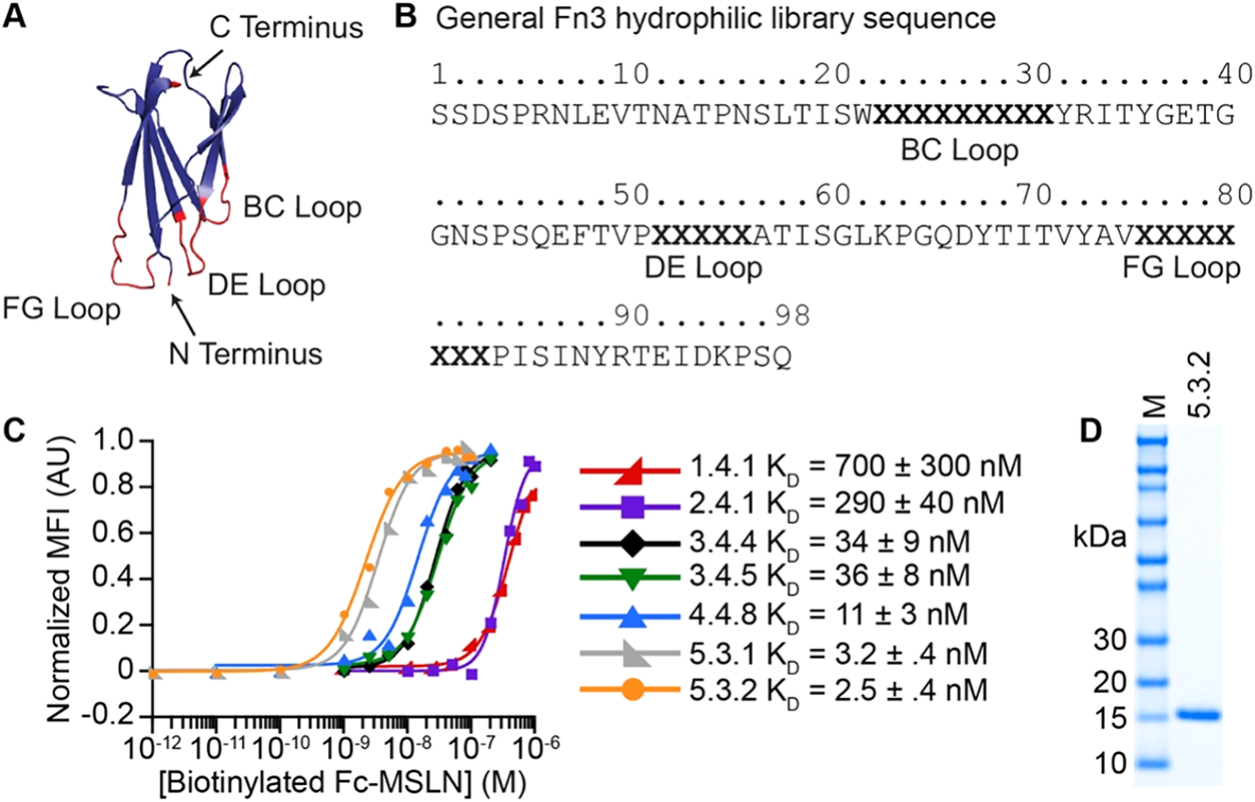
Identification of MSLN-binding variant Fn3 5.3.2. (A) The Fn3 scaffold has three solvent-exposed loops that form a binding interface for engineering interactions with target molecules (from PDB entry 1TTG). (B) Overall sequence of the Fn3 hydrophilic library. The BC, DE, and FG loops vary in length. Shown are the lengths that were predominantly selected during rounds of sorting. (C) Equilibrium binding assays to measure apparent dissociation constants for yeast-surface displayed Fn3 variants and soluble Fc-MSLN. Data for 1.4.1 and 2.4.1 modified from Sirois et al.^58^ Data for 3.4.4 and 3.4.5 modified from Sirois et al.^59^ (D) 5.3.2 was readily expressed and purified from bacterial culture.

### MSTO-211H-Derived Clonal Populations Exhibited a High Level of MSLN Expression

In pilot experiments with PM cells expressing MSLN at their natural levels, the maximum signal detected by flow cytometry for both MSLN expression and binding to Fn3 5.3.2 was low, and a larger dynamic range was needed for accurate measurements in equilibrium binding assays (data not shown). To better measure the binding affinity of Fn3 5.3.2 for MSLN, we first generated stably MSLN-overexpressing cells from the wild-type MSTO-211H cell line. We evaluated the MSLN expression on the cell surface through flow cytometry by incubating the cells with a rabbit anti-MSLN primary antibody and a goat anti-rabbit secondary phycoerythrin (PE)-conjugated antibody.

No MSLN expression was detectable on the wild-type MSTO-211H cell line (Figure 2), as no difference was observable between the fluorescence intensity of the untreated cells (black) and that of the cells treated with the MSLN detection antibodies (red). Among the clones derived from the clonal selection steps described in Experimental Procedures, two clonal populations, “MSTO clone 1” and “MSTO clone 7”, stably expressed high MSLN levels. MSTO clone 1 labeled with the detection antibodies showed a 27-fold increased fluorescence signal compared with the unlabeled control cells and a 12-fold increased signal compared to the wild-type MSTO-211H. MSTO clone 7 incubated with the detection antibodies showed a signal increase of 17-fold compared with unlabeled cells and 10-fold compared with wild-type MSTO-211H. Other viable clones evaluated did not exhibit any increase in the MSLN expression and were not employed for subsequent analysis.

**Figure 2:**
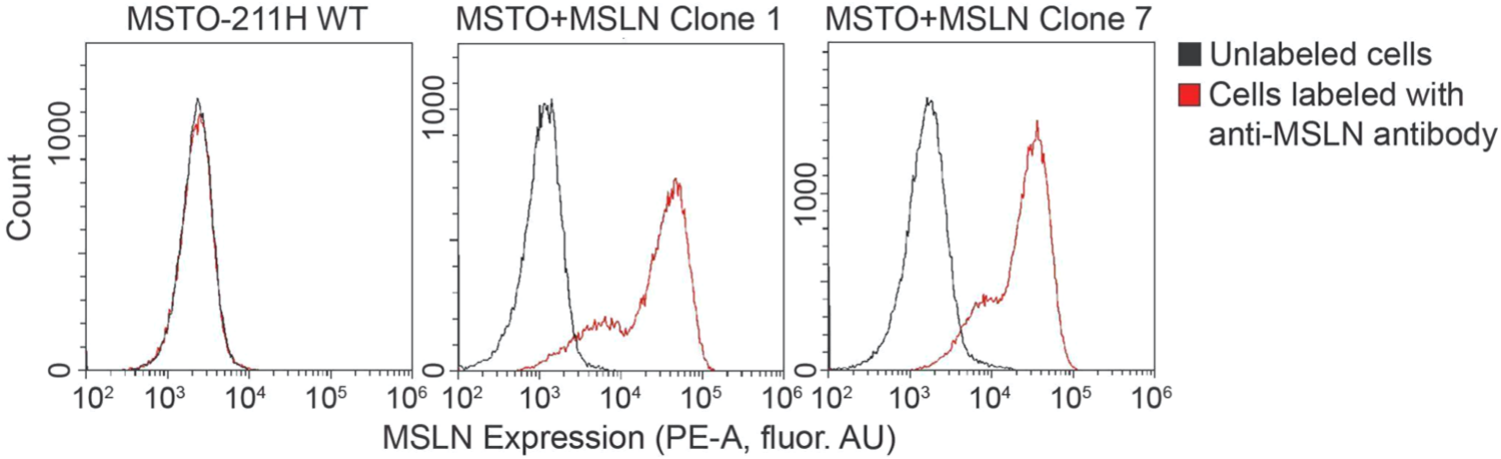
MSTO cell lines were generated with high levels of MSLN expression. Histograms showing MSLN expression in the MSTO-211H wild-type cell line (left) and the MSLN-overexpressing lines MSTO + MSLN clone 1 (center) and MSTO + MSLN clone 7 (right). The MSLN expression was evaluated through flow cytometry by incubating the cells with a rabbit anti-MSLN primary antibody and a goat anti-rabbit PE-conjugated secondary antibody (red). Unlabeled cells for each cell line (black) were used as a reference.

### Fn3 5.3.2 Binds MSLN-Overexpressing MSTO Clones with High Affinity

The binding affinity of Fn3 5.3.2 for MSLN was evaluated using the two MSLN-overexpressing clones, MSTO clone 1 and MSTO clone 7. Each cell line was incubated with a range of concentrations (0.015–1000 nM) of Fn3 5.3.2 with a hexahistidine tag, and the binding of Fn3 was detected through flow cytometry using an anti-HisTag antibody (Figure 3A). MSLN-negative cells MSTO-211H wild-type were employed as a negative control to exclude nonspecific binding between Fn3 5.3.2 and cell surface proteins other than MSLN. For MSTO clones 1 and 7, three replicates were carried out. We measured *K*_D_ = 11 ± 4 nM using MSTO clone 1 and *K*_D_ = 11 ± 2 nM for MSTO clone 7. We did not observe any binding between Fn3 5.3.2 and the MSLN-negative cell line MSTO-211H wild-type. These results highlighted a specific, tight binding affinity between Fn3 5.3.2 and MSLN.

**Figure 3:**
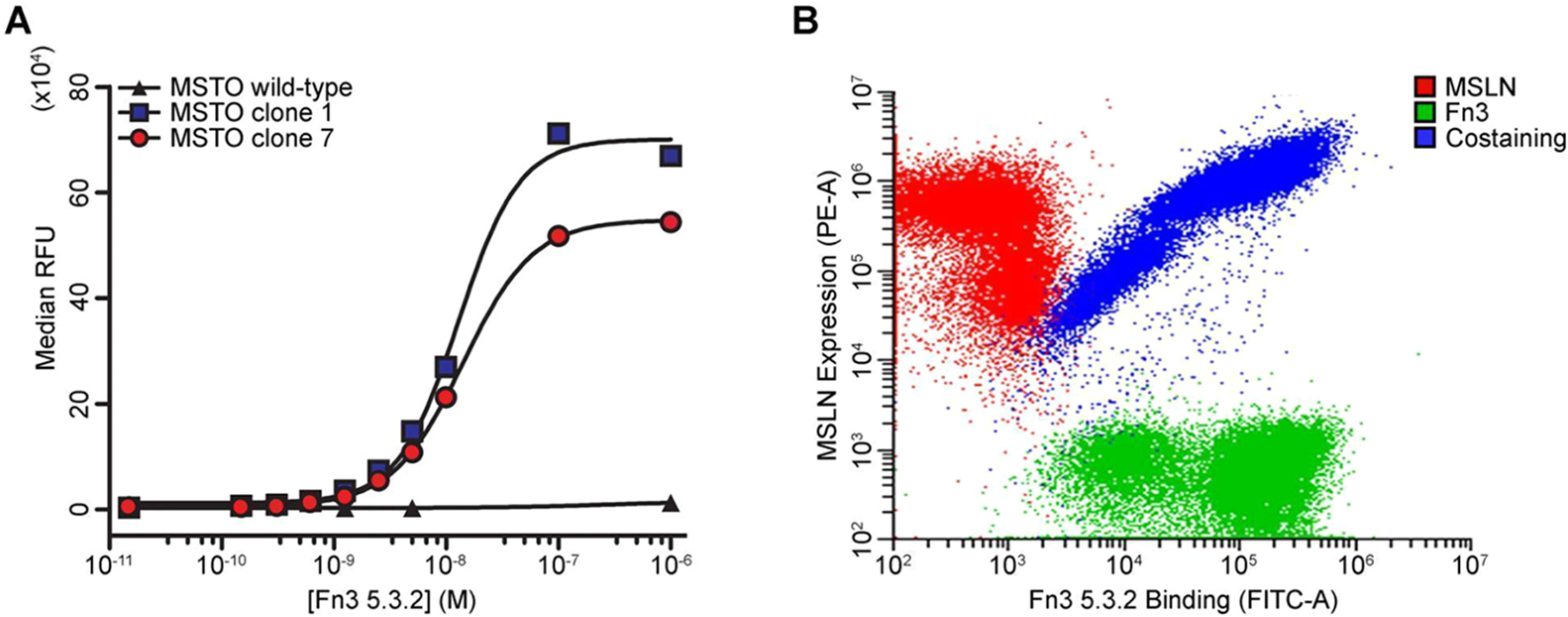
Fn3 5.3.2 binds MSLN-positive mesothelioma cell lines. (A) Representative binding curves for Fn3 5.3.2 using the MSLN-negative MSTO-211H wild-type and the MSLN-overexpressing lines MSTO clone 1 and MSTO clone 7. Each curve represents one of three experimental replicates. Data were fit to a sigmoidal curve, and *K*_D_ was calculated as the concentration of Fn3 5.3.2 yielding half of the maximum observed effect. MSTO clone 1: *K*_D_ = 11 ± 4 nM; MSTO clone 7: *K*_D_ = 11 ± 2 nM; MSTO WT: no binding detected. (B) Dot plot showing the results of the flow cytometry carried out on MSTO clone 1 stained with a PE-conjugated anti-MSLN primary antibody (red), Fn3 5.3.2 (10 nM), and an AF488-conjugated anti-HisTag antibody (green), or the combination of these two treatments (Costaining; blue). The *x*-axis reports the green fluorescence intensity detected through a fluorescein isothiocyanate (FITC) filter; the *y*-axis reports the red fluorescence intensity detected through a PE filter.

To further corroborate our observation, we incubated the MSTO clone 1 cell line with either a PE-conjugated anti-MSLN primary antibody (MSLN), Fn3 5.3.2 at a final concentration of 10 nM, and the FITC-conjugated anti-HisTag antibody (Fn3), or the combination of the two treatments (costaining). Each sample was evaluated through flow cytometry, and the fluorescence intensity detected in the PE and FITC channels was reported. The costaining sample showed a correlation between the MSLN and the Fn3 5.3.2 signal intensity that was not present in the control samples (MSLN or Fn3) (Figure 3B). These observations highlighted that Fn3 5.3.2 binding on the cell surface was proportional to MSLN expression, further supporting the specificity of MSLN/Fn3 binding.

### Fn3 5.3.2 and MSLN Colocalize and Internalize into MSLN-Positive MSTO Cell Lines

We visualized the localization of MSLN and Fn3 5.3.2 with an immunofluorescence assay. MSTO-7 cells were incubated with a combination of PE-conjugated anti-MSLN primary antibody, Fn3 5.3.2 with a hexahistidine tag at a final concentration of 100 nM, and an Fn3-detecting AF488-conjugated anti-HisTag antibody (costaining). To study how the MSLN/Fn3 complex behaves upon binding, cells were incubated at different temperatures (4 or 37 °C). The fluorescence signal was detected through confocal microscopy using a Red-Texas filter for MSLN (Figure 4A,D,G) and a FITC filter for Fn3 (Figure 4B,E,H). In samples incubated at 4 °C, staining for Fn3 5.3.2 and MSLN were colocalized on the surface of both cell lines (Figure 4C). Cells in the populations that did not express MSLN could be visualized by 4′,6-diamidino-2-phenylindole (DAPI) staining but had neither MSLN nor Fn3 5.3.2 staining, further confirming the specificity of the interaction of Fn3 5.3.2 with MSLN on the tumor cell surface. In samples incubated at 37 °C for 1 h, Fn3 5.3.2 staining revealed internalization in PM cells, supporting the findings of Sirois and colleagues^58^ (Figure 4E,F, red arrows). After 1 h of incubation, the PE-conjugated anti-MSLN antibody is mostly localized on the membrane of the cells, showing low or no internalization (Figure 4G,I). Since MSLN shedding is hypothesized to cause an “on target/off tumor” effect reducing therapeutic efficacy of antibody and CAR T-cell therapies,^60^ a drug carrier with rapid internalization, not limited by MSLN cleavage, could be a valuable strategy to improve the effectiveness of molecular imaging and targeted therapies. Moreover, Fn3 internalization in cancer cells paves the way for using radio-targeted alpha therapy against MSLN-positive tumors.^61^ Coupling Fn3 with alpha emitters could lower the damage to healthy tissues surrounding cancer cells.^62^ Preclinical and clinical studies have recently been reported using MSLN-targeted alpha-therapy, reporting high selectivity and efficacy in killing MSLN-positive tumor cells.^63,64^

**Figure 4:**
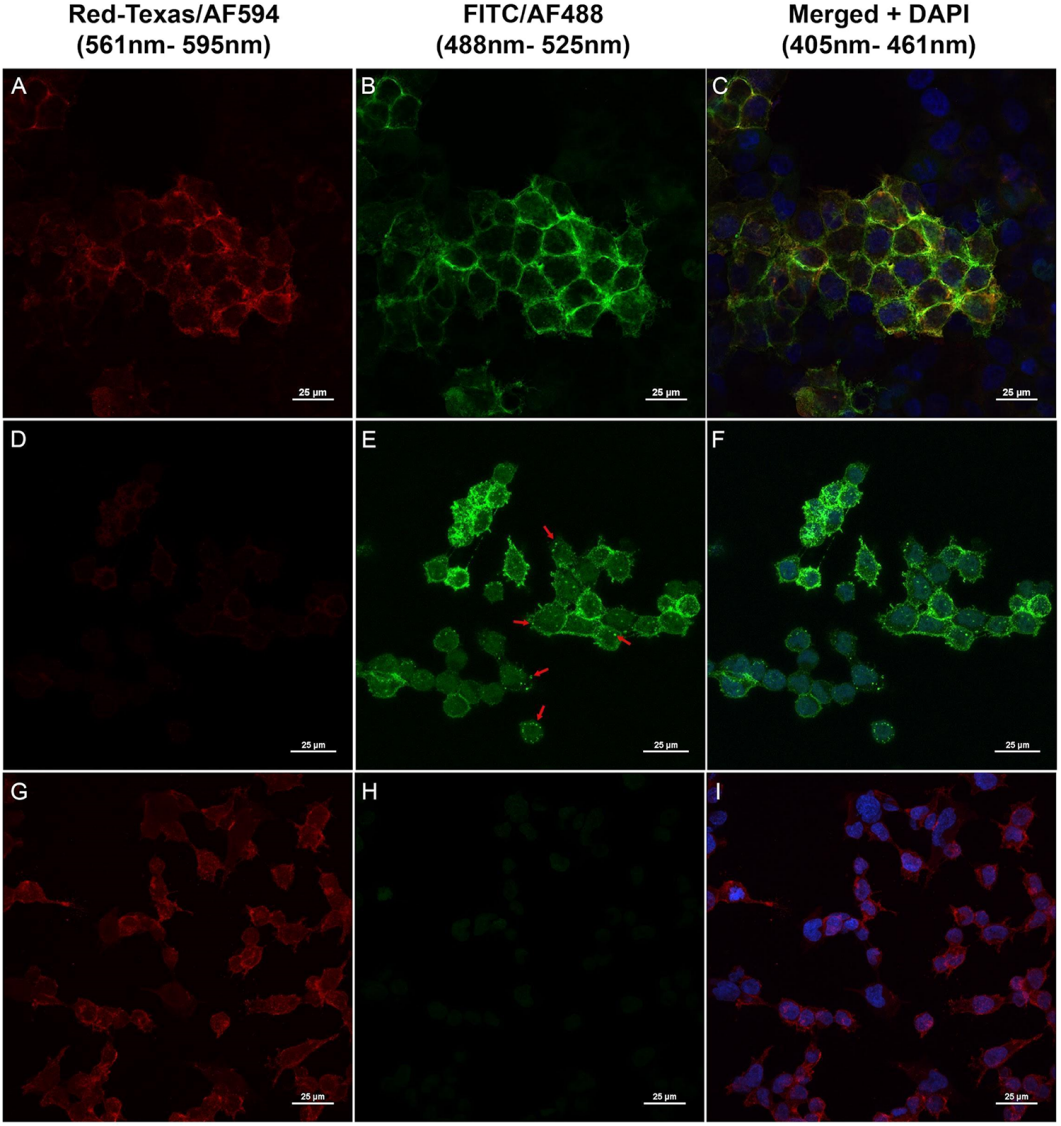
Fn3 5.3.2 and MSLN colocalize and internalize into MSLN-positive cells. MSLN-overexpressing MSTO clone 7 was evaluated using a Red-Texas filter (*E*_m_: 595 nm; *E*_x_: 561 nm; red) to detect MSLN (stained with a PE-conjugated primary antibody, A–G) and a FITC filter (*E*_m_: 525 nm; *E*_x_: 488 nm) to detect Fn3 (stained with an AF488-conjugated anti-HisTag antibody; B, E, H). DAPI (blue) was used to stain the nucleus (C, F, I). Panels (A–C) represent cells incubated at 4 °C for 1 h, in which colocalization of the MSLN/Fn3 complex on the cell surface is visible. Panels (D–F) show Fn3 internalization into punctate structures (red arrows) in PM cells, after 1 h of incubation at 37 °C. Panels (G–I) report cells treated with an anti-MSLN antibody for 1 h of incubation at 37 °C, mostly localized on the cancer cell surface.

### Fn3_cys_ 5.3.2 Functionalized with Radiometal Chelator DOTAGA and Coupled with ^69^Ga Maintains Binding to MSLN-Positive MSTO Cell Lines

In the initial work to conjugate a metal chelator to Fn3 5.3.2, we used primary amine chemistry to conjugate Fn3 5.3.2 to DOTAGA-anhydride. Fn3 5.3.2 bioconjugation with primary amine chemistry resulted in a mixture of different species, with varying degrees of labeling, characterized by different affinities for MSLN (Table S1 and Figure S1, Supporting Information). Therefore, we further modified Fn3 5.3.2 to enable site-specific thiol-maleimide chemistry. We designed an S97C mutation (Fn3_cys_ 5.3.2) in the C-terminus region of the scaffold, physically opposite the binding loops that recognize MSLN, avoiding steric hindrance that would likely occur with N-terminus modifications. Fn3 5.3.2 does not otherwise have any cysteine residues. In this way, we obtained a unique site for bioconjugation via thiol-maleimide chemistry. A Fn3_cys_-DOTAGA bioconjugate was synthesized with a 25-fold molar excess of maleimide-DOTAGA relative to Fn3_cys_ in phosphate-buffered saline (PBS) 1× and 5 mM TCEP. Electrospray ionization (ESI) mass spectrometry analyses showed ∼100% conversion with Fn3/DOTAGA in a 1:1 stoichiometry (Figure S2, Supporting Information).

The Fn3_cys_-DOTAGA conjugates were used to evaluate binding to MSLN on PM cell lines using the same protocol for equilibrium binding assays used for unconjugated Fn3 5.3.2. Fn3_cys_-DOTAGA maintained binding to MSLN with similar affinity as unconjugated Fn3 5.3.2 (*K*_D_ = 31 ± 2 nM, Figure 6A). Thus, S97C substitution and bioconjugation via thiol-maleimide chemistry resulted in an efficient strategy for chelator conjugation. The slight decrease in affinity could be due to increased flexibility, and therefore entropy, of the DOTAGA conjugate relative to the unconjugated Fn3, as well as to the potential for steric hindrance of the binding interface from the additional DOTAGA group.

After the confirmation of efficient chelator conjugation and low-nanomolar binding affinity for MSLN, Fn3_cys_-DOTAGA conjugates were coupled with ^69^Ga. In previous studies, ^68^Ga was employed to study the biodistribution of Fn3 scaffolds in mouse models, demonstrating high specificity and resolution using PET.^65,66^ We used the cold isotope for preliminary evaluation of Fn3_cys_-DOTAGA-^69^Ga potential for molecular imaging applications in the context of PM. Since coupling with radiometals requires low pH and high temperature, we evaluated the thermal stability of Fn3_cys_-DOTAGA at pH 4.5. With no ^69^Ga, the melting temperature (*T*_m_) was ∼65 °C (Figure 5). Incubating Fn3_cys_-DOTAGA at pH 4.5 with a 10-fold molar excess of ^69^Ga resulted in a similar stability (*T*_m_ ∼ 65.5 °C) (Figure 5). The *T*_m_ estimated when using Fn3_cys_-DOTAGA at pH 4.5 with a 100-fold excess of ^69^Ga decreased by 10 °C (*T*_m_ ∼ 55 °C) compared to Fn3_cys_-DOTAGA, suggesting that the ^69^Ga concentration used for coupling could affect protein stability at pH 4.5 (Figure 5).

**Figure 5:**
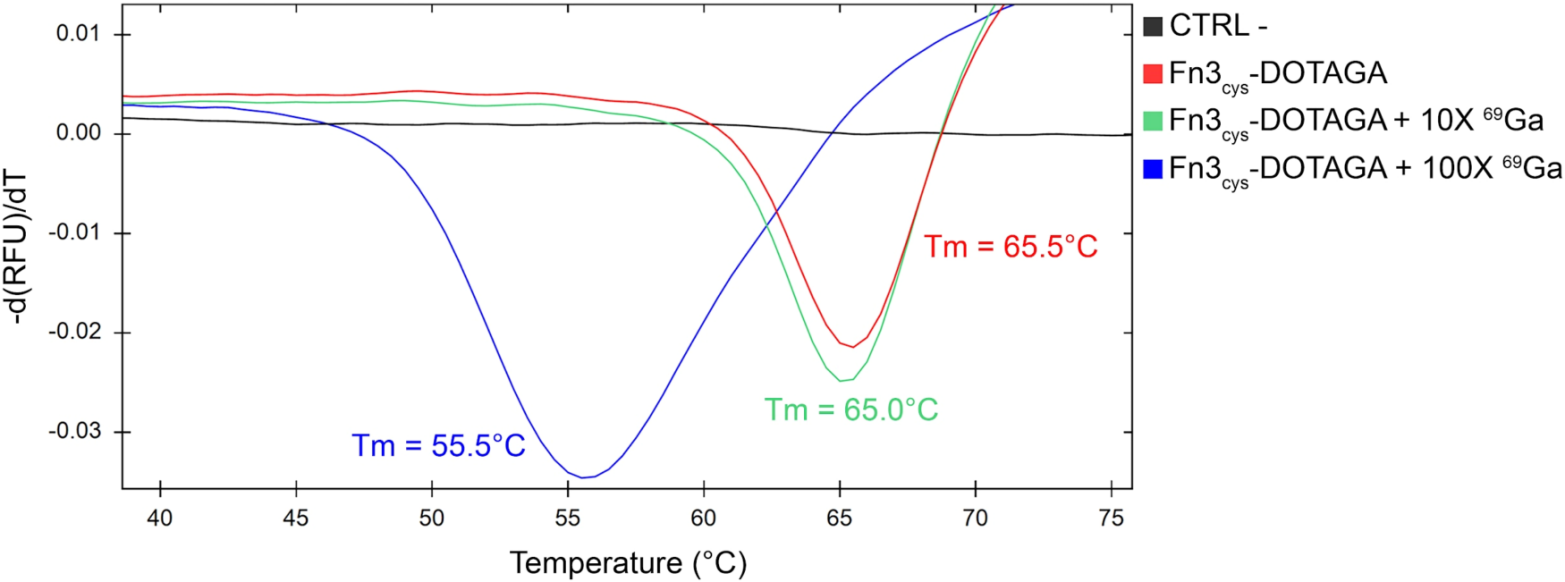
Fn3_cys_-DOTAGA-^69^Ga bioconjugates melting curves. Melting curves were carried out to explore the Fn3_cys_ stability at different temperatures in a solution at pH 4.5. This assay was performed to select the appropriate conditions for Fn3_cys_-DOTAGA and ^69^Ga coupling. The graph reports melting peaks of Fn3_cys_-DOTAGA at pH 4.5 without ^69^Ga (red), with 10× molar excess of ^69^Ga (green), and with 100× molar excess of ^69^Ga. A solution of 0.1 M sodium acetate and 0.1 M HCl at pH 4.5 was used as a negative control (black). The temperature selected for the subsequent coupling reaction was ∼5 °C below the estimated melting temperature.

Following thermal shift assays, the temperature selected for coupling was ∼5 °C below the estimated *T*_m_. We tested two labeling conditions, using a 10-fold or 100-fold molar excess of ^69^Ga, and assessed the coupling efficiency via ESI mass spectrometry, as described for Fn3_cys_-DOTAGA bioconjugation. When using a 10-fold molar excess for 15 min at 60 °C at pH 4.5, 60% of Fn3_cys_-DOTAGA was coupled with ^69^Ga. Using a 100-fold molar excess of ^69^Ga for 15 min at 50 °C at pH 4.5 resulted in 95% final conversion (Figure S2, Supporting Information). The Fn3_cys_-DOTAGA-^69^Ga resulting from this most favorable condition showed high affinity and specificity toward MSLN-positive cells (*K*_D_ = 15 ± 5 nM) (Figure 6B). The metal chelation appears to restore the affinity measured to that of the unconjugated Fn3, possibly due to decreasing the flexibility of the DOTAGA group and potentially reducing opportunities for steric hindrance.

**Figure 6:**
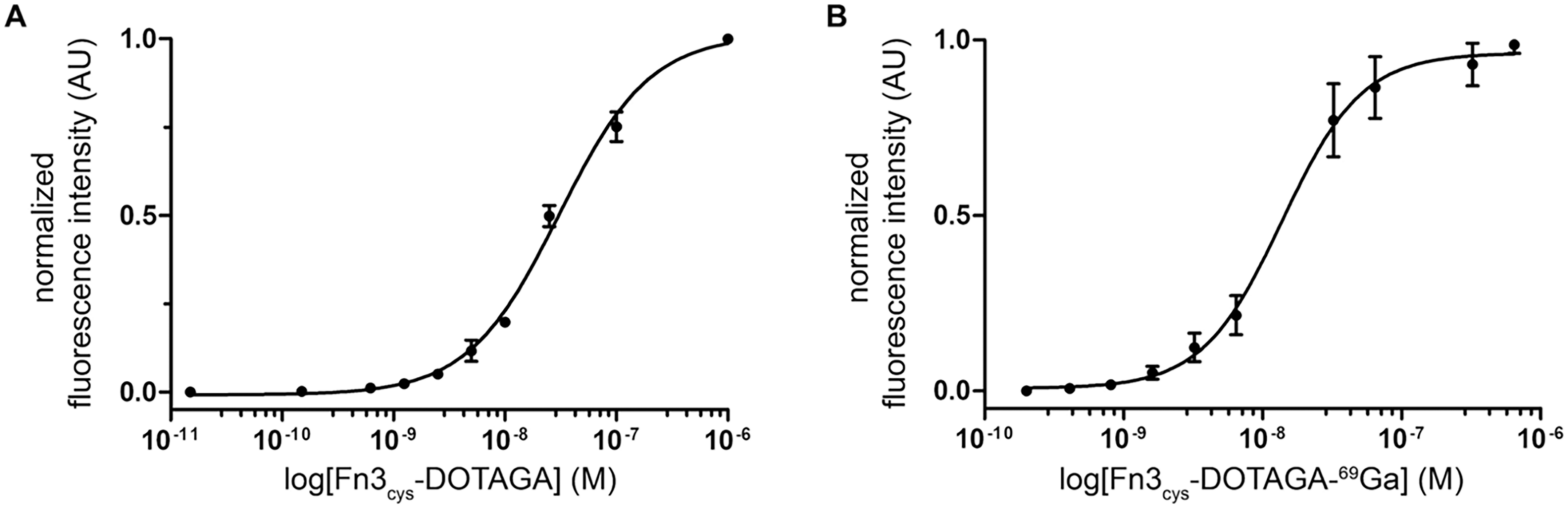
Fn3_cys_ functionalized with maleimide-DOTAGA and ^69^Ga binds MSLN-positive cells with high affinity. (A) Binding curves of three replicates for Fn3_cys_ 5.3.2 functionalized with maleimide-DOTAGA (A) and Fn3_cys_-DOTAGA-^69^Ga (B) using the MSLN-overexpressing line MSTO clone 7. Data from three replicates were normalized and fit to a sigmoidal curve. The binding affinity for each replicate was determined as the concentration yielding the half-maximum effect. For each concentration, mean (dot) and standard deviation (error bars) of the fluorescence intensity from the three replicates are represented in the graph (*K*_D_ = 31 ± 2 nM for Fn3_cys_-DOTAGA and *K*_D_ = 15 ± 5 nM for Fn3_cys_-DOTAGA-^69^Ga). An anti-His6 DyLight-488 antibody was used to detect the hexahistidine tag on the Fn3 conjugate.

## Conclusions

In conclusion, Fn3_cys_-DOTAGA-^69^Ga was engineered and validated as an MSLN-binding protein with relevance for theranostic applications with PM. Given these results, we are working to produce radiolabeled Fn3_cys_-DOTAGA-^69^Ga moieties and assess their biodistribution profile in animal models of PM. A recent publication has demonstrated the feasibility of molecular imaging targeting MSLN using a nanobody alternative protein scaffold in the context of ovarian cancer cell lines.^33^ Our current report broadens the scope of candidate molecular imaging agents targeting MSLN, with a focus on PM, including the application for radioligand therapy. Alongside previous data,^58^ the internalization of engineered Fn3 variants that bind MSLN suggests the inclusion of alpha-emitting radionuclides as molecules of interest for future studies.^34,67^ The shedding of MSLN from the tumor cell surface appears to be a factor in reducing the efficacy of MSLN-targeting therapeutic approaches with antibody and CAR T-cell approaches *in vivo*. The rapid internalization reported here of Fn3 5.3.2 compared to that of an anti-MSLN antibody suggests that these Fn3 variants may be internalized prior to target shedding from the cell surface. Future work will require a careful assessment of the extent to which MSLN shedding plays a role in the specific applications of molecular imaging and targeted radiotherapy *in vivo*. In summary, Fn3_cys_-DOTAGA-^69^Ga has the potential of enabling theranostic applications for PM and other MSLN-positive cancers, addressing the need for early imaging detection with improved prognosis, while opening a new opportunity to extend radioligand therapy to patients with PM.

## Experimental Procedures

No unexpected or unusually high safety hazards were encountered.

### Directed Evolution and Affinity Maturation of Fn3 Variants to Bind MSLN

A hydrophilic Gr2 Fn3 library (Figure 1A,B) previously evolved for MSLN-binding Fn3 variants was further affinity matured using yeast-surface display and directed evolution.^58,59,68–71^ For details, see the Supporting Information.

### Affinity Measurements Using Yeast-Surface Display

To identify lead Fn3 variants for soluble expression and further characterization, an equilibrium binding assay was performed with individual Fn3 variants displayed on the yeast surface and using soluble biotinylated Fc-MSLN to measure an apparent binding affinity. These measurements have been shown to align well with binding assays using soluble protein and human cell lines, and with *in vitro* measurements, such as surface plasmon resonance.^70^ Yeast displaying an identified Fn3 variant were incubated with a range of concentrations of biotinylated Fc-MSLN for 1 h at room temperature (RT) with gentle mixing and with a mouse 9E10 anti-c-myc antibody to detect full-length protein expression (1:50 dilution, 0.01 mg/mL, Fisher Scientific, 13-250-0). Yeast were washed with phosphate-buffered saline (PBS) with 0.1% w/v bovine serum albumin (PBSA), and incubated with streptavidin-AF488 and with goat-antimouse PE-conjugated secondary antibody for 30 min at 4 °C. Yeast were washed and immediately analyzed on a Guava flow cytometer (Millipore). Data were compensated for spectral overlap. Mean fluorescence intensity from binding of the expressing population was measured, plotted, and fit with a sigmoidal curve using Kaleidagraph (Synergy Software). The apparent *K*_D_ was determined as the concentration of soluble Fc-MSLN yielding the half-maximal signal. At least three replicates were measured, and the mean and standard deviation were reported.

### Fn3 Expression and Purification

MSLN-binding Fn3 variant 5.3.2 was prepared as previously described.^58,59^ For details, see the Supporting Information.

### Generation of Mesothelioma Cell Line with Enhanced MSLN Expression

MSLN-overexpressing clonal populations were established by starting from the MSLN-negative cell line MSTO-211H using a pcDNA3.1­(+)-based expression vector. The vector was purchased from Twin Helix (MSLN_pcDNA3.1) and harbored the coding sequence for the *MSLN* transcript variant 1 (accession number: NM_005823.6) and an upstream CMV constitutive promoter. The same vector also carried the *NeoR* gene, which provided resistance to the G418 antibiotic. MSLN_pcDNA3.1 (5 μg) was transfected into MSTO-211H cells using Lipofectamine 3000 (Invitrogen) following the manufacturer’s instructions. Six days after transfection, the G418 antibiotic was added and maintained in the culture medium at a final concentration of 500 μg/mL to select cells with stable expression of the *NeoR* gene. After 20 days of G418 selection, the MSLN expression was evaluated. To this end, 2 × 10^6^ cells were harvested using a nonenzymatic solution (0.02% EDTA in PBS) and incubated with either PBS (control) or a primary rabbit anti-MSLN antibody (1:50, 9 μg/mL; #196235, Abcam) for 1 h at 4 °C. The cells were then washed twice with a solution of PBSA and incubated with either PBS (control) or a secondary PE-conjugated goat anti-rabbit antibody (1:200, 2.5 μg/mL #72465, Abcam) for 30 min at 4 °C. Following this staining procedure, MSLN expression was evaluated through flow cytometry, and MSLN-positive cells were seeded at a single-cell density into 96-well plates using a BD FACSJazz cell sorter. The resulting viable clonal populations were re-evaluated for their MSLN levels following a similar staining procedure, and the clones exhibiting stable MSLN overexpression were selected for evaluating Fn3 5.3.2-MSLN-binding affinity.

### Cell Lines and Cell Culture

The MSTO-211H cell line was purchased from the American Type Culture Collection (ATCC, #CRL-2081). The cells were cultured in RPMI-1640 (#ECB2000L, Euroclone, S.p.A., Milan, Italy) supplemented with 10% fetal bovine serum (FBS, #10270-106, Gibco) and 1% penicillin–streptomycin (#ECB3001D, Euroclone, S.p.A., Milan, Italy). Cells were grown at 37 °C under a humidified atmosphere with 5% CO_2_.

### Equilibrium Binding Assays with Cancer Cell Lines

Wild-type MSTO and MSLN-overexpressing clones (1 and 7) were detached with 0.02% EDTA (no. E5134, Sigma-Aldrich) solution. For each sample, 3 × 10^5^ cells were pelleted at 400 g for 5 min at 4 °C; then, cells were washed with PBSA. Cells were incubated with a range of concentrations (0.015–1000 nM) of Fn3 5.3.2 in a total volume of 300 μL PBSA for 1 h at 4 °C with rotation. Cells were washed with PBSA and incubated with a mouse anti-His_6_ DyLight-488 antibody (1:50, 20 μg/mL, #117512, Abcam) in a total volume of 50 μL for 30 min at 4 °C with rotation and protection from light. MSLN expression was detected by a rabbit anti-MSLN antibody (1:50; 9 μg/mL, #196235, Abcam) and a goat anti-rabbit PE conjugate (1:200, 2.5 μg/mL, #72465, Abcam). After incubation, cells were washed with PBSA and analyzed using a CytoFLEX Flow Cytometer (Beckman Coulter). Data was fit to a sigmoidal curve. Dissociation constants (*K*_D_) were calculated as the Fn3 concentration, yielding half of the maximum signal for three replicates. Mean and standard deviation for *K*_D_ are shown. Following DOTAGA conjugation and ^69^Ga, binding assays with functionalized Fn3 were conducted similarly.

### Confocal Microscopy of Colocalization of Fn3 to the Membrane

MSLN-overexpressing MSTO clone 7 was seeded in a 6-well plate to achieve 80% confluency after 24 h, each well containing one coverslip. To study colocalization of Fn3 and MSLN, after 24 h, cells were fixed with 4% paraformaldehyde (PFA) for 15 min at RT, and washed twice with PBSA for 5 min in oscillation. Cells were blocked with 4% horse serum for 1 h at RT, and washed with PBSA. For MSLN staining, cells were treated with 50 μL of PE-conjugated primary anti-MSLN antibody (1:200, 2.5 μg/mL, #ab252136 Abcam) and incubated for 1 h at 4 °C. To evaluate the binding between MSLN and Fn3, cells were treated with 100 nM of Fn3 5.3.2 in a total volume of 50 μL and incubated for 1 h at 4 °C. Costaining was performed by incubating cells with both treatments described above. Then, cells were washed three times with PBSA for 5 min in oscillation. For MSLN-Fn3 binding samples and costaining, cells were treated with a mouse anti-His_6_ DyLight-488 antibody (1:100, 10 μg/mL, #117512, Abcam) in a total volume of 50 μL, and incubated for 1 h at 4 °C and washed as before. Cell nuclei were stained using DAPI (1:1000, ThermoFisher) and incubated for 5 min at RT, protected from light, and washed twice with PBSA. The control sample was incubated with a mouse anti-His6 DyLight-488 antibody (1:100, 10 μg/mL, #117512, Abcam) without Fn3, under the same conditions. Coverslips were mounted on microscope slides with mounting medium (no. F4680, Sigma-Aldrich) and sealed with nail polish. Samples were observed by using a Nikon A1+ confocal microscope.

### Confocal Microscopy of Internalization of Fn3/MSLN in Cancer Cells

To study the internalization of the MSLN/Fn3 5.3.2 complex into PM cancer cells, MSTO clone 7 was treated as described before. Briefly, MSTO clone 7 cells were seeded in a 6-well containing one coverslip. When the cells reached 80% confluence (after 48 h), cells were treated with PE-conjugated primary anti-MSLN antibody (1:200, 2.5 μg/mL, #ab252136 Abcam) or with 100 nM of Fn3 5.3.2 in a total volume of 50 μL, and incubated for 1 h at 37 °C. After washing with PBSA for 5 min in oscillation, cells were fixed with 4% paraformaldehyde (PFA) for 15 min at RT, and washed twice with PBSA for 5 min in oscillation. Cells were permeabilized with 1 mL of Triton 0.25% for 5 min at RT and washed twice with PBSA while oscillating. The blocking step with HS 4%, incubation with anti-His_6_ DyLight-488 antibody (1:100, 10 μg/mL, #117512, Abcam), and nuclei staining with DAPI were carried out as described in the previous section. Coverslips were mounted on microscope slides, as described previously. Samples were analyzed by using a Nikon TI2 confocal microscope. To evaluate the spatial arrangement of MSLN/Fn3 internalization, z-stack imaging was used (0.2 μm steps, range ∼10 μm).

### Fn3 5.3.2 Further Engineering to Optimize Bioconjugation with DOTAGA

As a first attempt to enable radiolabeling of Fn3 5.3.2, the protein was functionalized with DOTAGA-anhydride, exploiting the primary amine at the N-terminus of the scaffold. Fn3 5.3.2 bioconjugation was executed with modifications of procedures described by Moreau and colleagues.^72^ The optimal condition for bioconjugations is monolabeling, i.e., 1:1 DOTAGA/antibody stoichiometry, which would ensure more reproducible radiolabeling and facilitate use in *in vitro* and *in vivo* models.^73–75^ To this end, multiple conditions were tested to find the optimal pH and DOTAGA-molar excess, allowing the 1:1 stoichiometry between the protein and the chelator. However, considering the Fn3 5.3.2 amino acid sequence, five primary amines are potentially available for bioconjugation with DOTAGA-anhydride, including the N-terminus and four lysine residues. This is reflected in the presence, in all reaction conditions tested, of products with a range of degrees of labeling (Table S1 and Figure S1, Supporting Information).

Fn3-based scaffolds do not include any cysteine or disulfide bonds in their structure, allowing the introduction of a unique cysteine for functionalization and radiolabeling.^65^ Therefore, Fn3 5.3.2 was further modified by replacing serine 97 with cysteine (Fn3_cys_ 5.3.2). In this way, a unique cysteine was available for bioconjugation with maleimide-DOTAGA, via thiol-maleimide chemistry. A gene block with the Fn3_cys_ 5.3.2 nucleotide sequence was designed with the S97C substitution (Eurofins Genomics https://eurofinsgenomics.eu/). After cloning the Fn3_cys_ 5.3.2 gene into the pET vector and transformation into DH5α *Escherichia coli* cells (MAX Efficiency DH5α Competent Cells, no. 18258012, Invitrogen), the amino acid substitution was confirmed by Sanger sequencing. Fn3_cys_ 5.3.2 was expressed and purified as described above. Buffer exchange into PBS with 5 mM tris­(2-carboxyethyl)­phosphine (TCEP, Merck, #75259) and protein concentration was carried out with Amicon Ultra centrifugal filtration devices (MWCO 10 kDa, #UFC801024, Merck). Purity of the protein sample was analyzed by SDS-PAGE on a BioRad ChemiDoc MP imaging system.

### DOTAGA Conjugation and Conjugate Characterization

Fn3_cys_ was functionalized with maleimide-DOTAGA, using a 25-fold molar excess of chelator with respect to Fn3_cys_. A volume of 1.5 mL of a 7 mg/mL suspension of maleimide-DOTAGA (Chematech, Dijon, France) in anhydrous chloroform (Carlo Erba, Val de Reuil, France) was pipetted under ultrasonication and aliquoted into 1.5 mL polypropylene microtubes. The chloroform was then evaporated under a gentle flow of nitrogen. Subsequently, 500 μL of purified Fn3_cys_ (1 mg/mL, 0.50 mg, 39 nmol, 1 equiv) in PBS 1× and 5 mM TCEP (pH 7.4) were added to a maleimide-DOTAGA aliquot corresponding to 25 equiv (0.586 mg, 975 nmol). The solution was gently mixed, incubated at RT for 1 h, and stored at 4 °C. Unbound DOTAGA was removed using PD-10 desalting columns (#GE17-0851-01, Cytiva), following the manufacturer’s instructions. Then, the protein was lyophilized and stored at −20 °C until use. Fn3-DOTAGA conjugation efficiency was evaluated through electrospray ionization (ESI) mass spectrometry (ThermoFisher Scientific Orbitrap Exploris 120). Before injection into the mass spectrometer, conjugated and nonconjugated protein samples were diluted in a solution of 50% acetonitrile, 0.1% formic acid, and ultrapure water.^76^ Acquisition range was set between 800 and 3000 *m*/*z*, and quantification was performed on the 14+ cluster. The conjugation efficiency was evaluated considering the relative intensity of the *m*/*z* peaks of the conjugate and of the native protein (12,833 Da). This protocol was applied to determine the percentage of functionalized protein, assuming a similar ionization efficiency of the two compounds. Then, the binding of Fn3_cys_-DOTAGA to MSLN was evaluated as described above using an equilibrium cell binding assay.

### Protein Thermal Shift Assays

To assess the stability of Fn3_cys_ at high temperature and pH 4.5, which are used in radiolabeling conditions, a melting curve of the protein was carried out using ProteOrange (#40210, LumiProbe). Briefly, Fn3_cys_-DOTAGA (0.2 mg/mL) was reconstituted in a solution at pH 5.2 of 0.1 M HCl and 0.5 M sodium acetate (NaOAc). Samples were prepared in optically clear PCR tubes (0.2 mL). ^69^GaCl_3_ (analog of radioisotope ^68^Ga) was purchased from ThermoFisher Scientific (#444100250), weighed, and resuspended in HCl 0.1 M. A molar excess of 10-fold or 100-fold ^69^GaCl_3_ with respect to Fn3_cys_-DOTAGA was added to protein samples to reach a final concentration of 0.1 mg/mL Fn3_cys_-DOTAGA (2 μg in 20 μL) and pH 4.5. Fn3_cys_-DOTAGA reconstituted in NaOAc (0.1 M) and HCl (0.1 M) (pH 4.5) was used as a control. For each condition, two replicates were carried out. ProteOrange working solution 20× was added (1 μL) to each sample. To estimate the melting temperature, samples were incubated in the CFX96 Touch Real-Time PCR Detection System (BioRad). The thermocycling protocol consisted of temperatures varying from 20 to 90 °C in increments of 0.5 °C for 10 s.

### Synthesis and Characterization of Fn3_cys_-DOTAGA-^69^Ga

We coupled Fn3_cys_-DOTAGA to the cold metal Gallium-69 (Ga69), as an alternative to the widely used radioisotope ^68^Ga.^77,78^ ^69^GaCl_3_ was purchased from ThermoFisher Scientific (#444100250), weighed, and resuspended in HCl 0.1 M. For the coupling reaction, Fn3_cys_-DOTAGA 2 mg/mL (1 mg, 78 nmol) was resuspended in 0.5 mL in a solution of NaOAc 0.5 M and HCl 0.1 M (pH 5.2). ^69^GaCl_3_ in HCl 0.1 M was added to the protein sample in 10- (780 nmol, 0.5 mL) or 100-fold (7800 nmol, 0.5 mL) molar excess, leading to a labeling solution at pH 4.5. Fn3_cys_-DOTAGA samples were incubated at 60 °C (10× ^69^GaCl_3_) or 50 °C (100× ^69^GaCl_3_) for 15 min with gentle shaking in a thermo-mixer. Fn3_cys_ was incubated under the same conditions and was used as a control. Unbound ^69^GaCl_3_ was removed using PD-10 desalting columns (#GE17-0851-01, Cytiva), following the manufacturer’s instructions. Then, the conjugate was lyophilized and stored at −20 °C until use. Fn3_cys_-DOTAGA-^69^Ga conjugation efficiency was evaluated through ESI mass spectrometry (ThermoFisher Scientific Orbitrap Exploris 120), as described in the previous section. After identifying the best coupling condition, the binding affinity of Fn3_cys_-DOTAGA-^69^Ga toward MSLN was measured via flow cytometry, as described above.

## Supplementary Material



## Notes

Table of mass spectrometry data for all DOTAGA conjugation reaction conditions (Table S1); Fn3 5.3.2-(DOTAGA-anhydride) binding affinity curve (Figure S1); mass spectrometry of Fn3_cys_-DOTAGA-^69^Ga conjugates (Figure S2); experimental procedures (PDF)

## Abbreviations

AF: Alexa Fluor
DAPI: 4′,6-diamidino-2-phenylindole
DOTAGA: 1,4,7,10-tetraazacyclododecane,1-(glutaric acid)-4,7,10-triacetic acid
FACS: fluorescence-activated cell sorting
FITC: fluorescein isothiocyanate
Fn3: 10th domain of fibronectin type III
IPTG: isopropyl-*b*-d-thiogalactopyranoside
MACS: magnetic-activated cell sorting
MSLN: mesothelin
MW: molecular weight
PBS: phosphate-buffered saline
PBSA: phosphate-buffered saline, with 0.1% (w/v) bovine serum albumin
PE: phycoerythrin
PFA: paraformaldehyde
PM: pleural mesothelioma
