# Supplementary material for: Development of a Mesothelin-Binding Engineered Scaffold Protein as a Theranostic for Pleural Mesothelioma
Source: Bioconjug Chem. 2025 Oct 27;36(11):2436–47. doi: 10.1021/acs.bioconjchem.5c00425 (PMC12635973; doi:10.1021/acs.bioconjchem.5c00425)
Supplement: Supplementary file 1 [file bc5c00425_si_001.pdf]

SUPPORTING INFORMATION

Development of a Mesothelin-Binding Engineered Scaffold Protein as a Theranostic for Pleural Mesothelioma

Roberto Silvestri,[1,†] Margherita Piccardi,[1,2,†], Alessia Laurenza,[1] Filomena Rea,[4] Allison R. Sirois,[2,3] Martina Lari,[1] Francesco Bartoli,[4] Giovanni Signore,[1] Lorena Tedeschi,[6] Elisabetta Ferraro,[1] Paola Anna Erba,[5] Roberto Giovannoni,[1] Stefano Landi,[1] Federica Gemignani,[1 ‡] Sarah J. Moore [2,3 ‡ *]

† R.S. and M.P. have contributed equally to this work and share first authorship.
‡ F.G. and S.J.M. have contributed equally to this work and share last authorship.

*Corresponding author:
Sarah J. Moore
E-mail: sjmoore@smith.edu

AUTHOR ADDRESSES

1. Department of Biology, University of Pisa, Pisa, Italy, 56126
2. Picker Engineering Program, Smith College, Northampton, Massachusetts, United States, 01063
3. Molecular and Cellular Biology Program, University of Massachusetts Amherst, Amherst, Massachusetts, United States, 01003
4. Department of Translational Research and New Technologies in Medicine and Surgery, University of Pisa, Pisa, Italy, 56126
5. School of Medicine and Surgery, University of Milan Bicocca, Milan, Italy, 20126
6. Institute of Clinical Physiology, CNR, Pisa, Italy, 56124

Table S1. Mass spectrometry of Fn3 5.3.2-(DOTAGA-anhydride) conjugates.

Conjugation conditions		Species[a]	Estimated MW (Da)[b]	Composition (%)[c]
pH	DOTAGA excess			
7.0	5X	Fn3	12816.4	44.0
		Fn3-(1)DOTAGA	13274.6	46.3
		Fn3-(2)DOTAGA	13732.8	9.7
		Fn3-(3)DOTAGA	-	0.0
	50X[d]	Fn3	12816.3	9.0
		Fn3-(1)DOTAGA	13275.5	31.7
		Fn3-(2)DOTAGA	13733.7	41.1
		Fn3-(3)DOTAGA	14191.9	18.2
	100X	Fn3	12816.3	13.8
		Fn3-(1)DOTAGA	13275.5	43.4
		Fn3-(2)DOTAGA	13732.7	33.3
		Fn3-(3)DOTAGA	14191.9	9.5
7.5	5X	Fn3	12816.4	34.0
		Fn3-(1)DOTAGA	13275.6	11.0
		Fn3-(2)DOTAGA	13732.8	25.8
		Fn3-(3)DOTAGA	14192.0	29.2
	50X	Fn3	12816.3	11.3
		Fn3-(1)DOTAGA	13275.5	13.6
		Fn3-(2)DOTAGA	13732.7	40.3
		Fn3-(3)DOTAGA	14191.9	34.7
	100X	Fn3	12816.3	26.6
		Fn3-(1)DOTAGA	13274.5	41.3
		Fn3-(2)DOTAGA	13732.7	23.0
		Fn3-(3)DOTAGA	14193.0	9.0
8.0	5X	Fn3	12816.4	29.4
		Fn3-(1)DOTAGA	13274.6	41.9
		Fn3-(2)DOTAGA	13732.8	22.6
		Fn3-(3)DOTAGA	14193.0	6.1
	50X	Fn3	12814.3	5.3
		Fn3-(1)DOTAGA	13275.5	23.1
		Fn3-(2)DOTAGA	13732.7	41.4
		Fn3-(3)DOTAGA	14191.9	30.2
	100X	Fn3	12816.3	36.7
		Fn3-(1)DOTAGA	13275.5	40.0
		Fn3-(2)DOTAGA	13733.7	15.3

Conjugation conditions		Species[a]	Estimated MW (Da)[b]	Composition (%)[c]
pH	DOTAGA excess			
7.0	5X	Fn3	12816.4	44.0
		Fn3-(1)DOTAGA	13274.6	46.3
		Fn3-(2)DOTAGA	13732.8	9.7
		Fn3-(3)DOTAGA	-	0.0
	50X[d]	Fn3	12816.3	9.0
		Fn3-(1)DOTAGA	13275.5	31.7
		Fn3-(2)DOTAGA	13733.7	41.1
		Fn3-(3)DOTAGA	14191.9	18.2
	100X	Fn3	12816.3	13.8
		Fn3-(1)DOTAGA	13275.5	43.4
		Fn3-(2)DOTAGA	13732.7	33.3
		Fn3-(3)DOTAGA	14191.9	9.5
		Fn3-(3)DOTAGA	14193.0	8.0

[a] Data were retrieved by ESI mass spectrometry analyzing Fn3 conjugated in triethylammonium bicarbonate at various pHs (7.0, 7.5, 8.0) with 5X-/50X-/100X- DOTAGA-anhydride molar excess.

[b] Estimated molecular weight (MW) of Fn3, or Fn3 mono-/bi-/tri-functionalized with DOTAGA-anhydride (MW = 458.46 Da). Expected MWs for each species are: Fn3 (12816.3 Da), Fn3-(1)DOTAGA (13275.5 Da), Fn3-(2)DOTAGA (13733.7 Da), Fn3-(3)DOTAGA (14191.9 Da).

[c] The percentage of each species is shown.

[d] Bioconjugation at pH 7.0 with 50X DOTAGA-anhydride molar excess was selected as optimal condition for further binding assays.

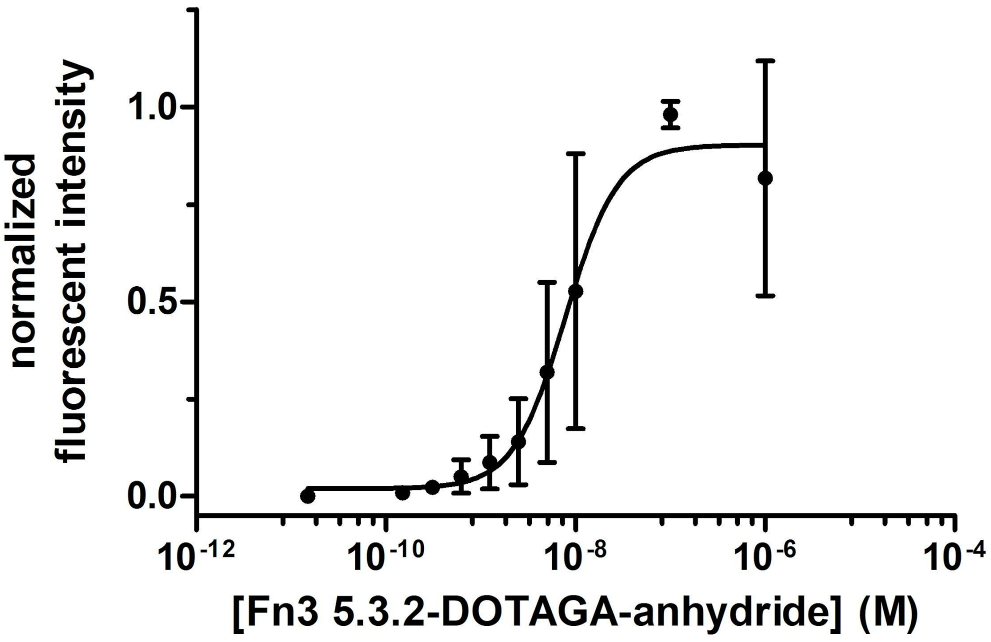

Figure S1. Fn3 5.3.2-(DOTAGA-anhydride) binds MSLN+ cells with high variability.

Equilibrium binding curve for Fn3 5.3.2 functionalized with DOTAGA-anhydride using the MSLN-overexpressing line MSTO clone 7. Data from each of three independent replicates were fit to a sigmoidal curve. The binding affinity for each replicate was determined as the concentration yielding the half maximum effect. Mean ± standard deviation of three dissociation constants: K_D = 12 ± 11 nM. Each data point was normalized and mean (black dot) and standard deviation (error bars) are plotted above.

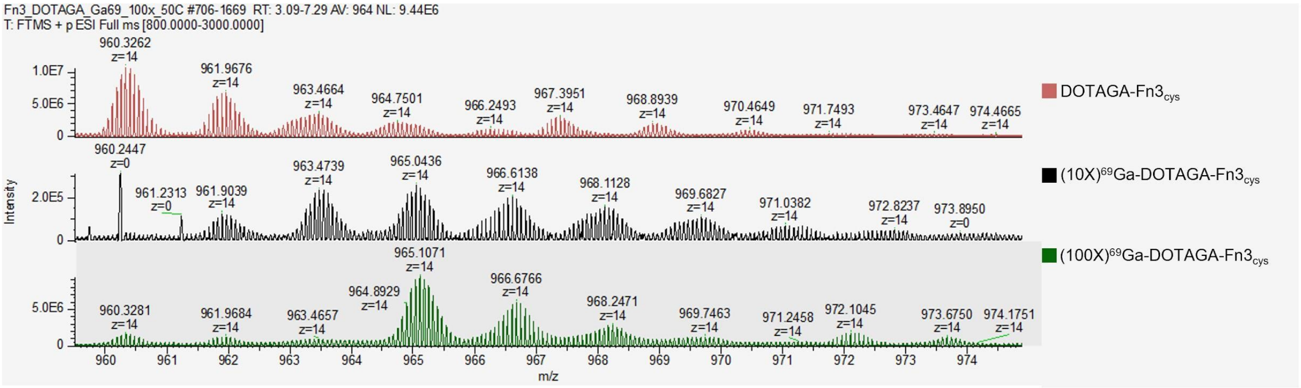

Figure S2. Mass spectrometry of Fn3$_{cys}$-DOTAGA-^{69}Ga conjugates.

Data were retrieved by ESI mass spectrometry analyzing Fn3 conjugated in sodium acetate 0.1M and HCl 0.1M at pH 4.5 with 10X- or 100X- ^{69}Ga molar excess (black and green spectra, respectively). Fn3$_{cys}$-DOTAGA spectrum is represented in red. The mass to charge ratio (m/z) is shown on the x-axis. The intensity of the signal is reported on the y-axis. Quantification was performed on the 14+ cluster. The m/z peak corresponding to Fn3$_{cys}$-DOTAGA is ~960.3, while m/z ~965.1 indicates Fn3$_{cys}$-DOTAGA-^{69}Ga. The multiple peaks revealed a mass increment consistent with one or multiple Na^+ adducts to Fn3$_{cys}$. Specifically, in black (10X) and green (100X) spectra, m/z ranging from 960 to 963.5 indicates the presence of Fn3$_{cys}$-DOTAGA and Na^+ adducts. Values of m/z starting from 965.1 correspond to a mass increment consistent with the addition of ^{69}Ga and/or multiple Na^+ adducts per each Fn3$_{cys}$-DOTAGA molecule.

Supporting Information Experimental Procedures

Directed evolution and affinity maturation of Fn3 variants to bind MSLN

A hydrophilic Gr2 Fn3 library previously evolved for MSLN-binding Fn3 variants was further affinity matured using yeast surface display and directed evolution. Error-prone PCR with nucleotide analogs was used to add diversity to binding loops and the overall protein framework to generate a fourth generation library (5.5 x 10^7 transformants). The library was sorted twice by magnetic-activated cell-sorting (MACS) using biotinylated, Fc-tagged recombinant human MSLN (Acro Biosystems #MSN-H826x) followed by a fluorescence-activated cell sorting (FACS) selection for full-length clones using an antibody against the C-terminal c-myc epitope tag. Full-length clones were incubated with a chicken anti-c-myc antibody and the biotinylated Fc-tagged MSLN. To increase the sorting stringency, concentrations of MSLN were decreased over four iterative rounds of enrichment, with reducing concentrations of MSLN for each round to increase sort stringency. Cells were washed and incubated with a goat anti-chicken Alexa Fluor-647 (AF647) conjugate and AF488-conjugated streptavidin. Cells were washed and double-positive yeast cells were collected on a BD BioSciences FACSAria II. A sort window was created collecting 0.1-1% of cells, sorting on a diagonal by collecting those yeast that exhibited the greatest MSLN binding for a given expression level. The number of cells sorted was at least ten times the size of the present library at that sort, such that each unique variant should be sampled. Plasmid DNA from the enriched fourth generation library was recovered using a Zymoprep Yeast Plasmid Miniprep II kit (Zymo Research) following manufacturer's protocol, isolating DNA from the pooled yeast population, collecting DNA from sufficient number of cells to have 10-fold coverage of the smallest collected population during sorting, such that all unique plasmid sequences would be statistically likely to be sampled. The population DNA was transformed into XL1-Blue bacteria (5 µl of Zymoprep DNA, typical yield 1.25x10^7 plasmids/µl, for each 50 µl of bacteria) and plated onto selective media to isolate colonies with unique plasmids. Plasmids from individual bacterial clones were sequenced by standard Sanger DNA sequencing methods. Batches of additional plasmids were sequenced until no new sequences emerged. Unique plasmids were then individually transformed into EBY100 yeast to enable binding measurements with individual protein sequences. A fifth-generation library (4.4 x 10^7 transformants) was generated from the enriched, pooled fourth generation DNA, and similarly sorted using MACS and FACS, with a final third FACS sort using 1 nM of biotinylated Fc-tagged MSLN. Unique plasmid sequences were determined as described for the fourth-generation library.

Fn3 expression and purification

MSLN-binding Fn3 variant 5.3.2 was prepared as previously described.[1,2] Briefly, Fn3 gene was cloned into a pET vector with a C-terminal hexahistidine tag and expressed in BL21(DE3) *E. coli*. Cultures were grown in LB with kanamycin (50 µg/mL) and induced overnight at 20°C with 0.5 mM isopropyl-b-D-thiogalactopyranoside (IPTG). Cells were resuspended in lysis buffer (35 mM Na_2HPO_4×dibasic, 15 mM NaH_2PO_4×monobasic, 500 mM NaCl, 5 mM CHAPS, 25 mM imidazole, 5% glycerol) supplemented with an EDTA-free protease inhibitor (Pierce), and lysed by repeated freezing and thawing. Soluble fractions were isolated by centrifugation. Fn3 variants were purified by cobalt affinity chromatography with HisPur cobalt resin (Thermo Fisher). Protein samples were dialyzed into water, lyophilized, reconstituted with 1X PBS to the desired

concentration, and analyzed for purity by SDS-PAGE on a BioRad ChemiDoc MP imaging system.

Supporting Information References

1. Sirois AR, Deny DA, Baierl SR, George KS, Moore SJ. Fn3 proteins engineered to recognize tumor biomarker mesothelin internalize upon binding. *PLoS ONE* **2018**, 13:e0197029.

2. Sirois AR, Deny DA, Li Y, Fall YD, Moore SJ. Engineered Fn3 protein has targeted therapeutic effect on mesothelin-expressing cancer cells and increases tumor cell sensitivity to chemotherapy. *Biotechnol Bioeng* **2020**, 117:330–341.
